# Changes in Maternal Glucose Metabolism after the Administration of Dexamethasone for Fetal Lung Development

**DOI:** 10.1155/2012/652806

**Published:** 2012-03-25

**Authors:** Xu Jian Yun, Liang Zhaoxia, Chai Yun, Fang Qin, Chen Yuanyuan, Chen Danqing

**Affiliations:** Obstetrical Department, Women's Hospital, School of Medicine, Zhejiang University, Hangzhou 310006, China

## Abstract

*Aims*. Antenatal dexamethasone administration for fetal lung development may impair maternal glucose tolerance. In this study, we investigated whether glucose and insulin levels differed among singleton and twin pregnancies and pregnancies with impaired glucose tolerance (IGT) after treatment with dexamethasone. *Methods*. Singleton pregnancies, twin pregnancies, and pregnancies with IGT between 28 and 33 weeks of gestation whose mothers were treated with dexamethasone were enrolled in this study. Exclusion criteria included gestational hypertension, diabetes, renal disorders, and infectious diseases. The fasting plasma glucose and insulin levels were checked before administration and 24 h, 48 h, and 72 h after treatment was completed. *Results*. Mean glucose levels were significantly higher in the twin pregnancy and IGT groups at 24 h and 48 h after the administration of dexamethasone than those in the singleton pregnancy group (*P* < 0.05). Although there was no significant difference in glucose levels before administration and 72 h after dexamethasone administration among the three groups, insulin levels in the IGT group were significantly higher (*P* < 0.05). Insulin levels in the singleton pregnancy group at 24 h and 48 h after treatment were significantly lower than in the twin and IGT groups. *Conclusion*. The effects on maternal fasting blood glucose and insulin levels of dexamethasone administrated to promote fetal lung maturation correlated with embryo number and the presence of IGT.

## 1. Introduction

The most common cause of deaths among preterm babies is respiratory distress syndrome (RDS); antenatal corticosteroid (ACS) treatment for pregnant women at risk of preterm birth is an established intervention for the prevention of RDS. Liggins and Howie first described this indication in 1972, when they demonstrated that ACS could reduce the risk of neonatal RDS from 25.8% to 9.0%, and the rate of neonatal mortality dropped from 15.0% to 3.2% [1, 2]. In 1994, a National Institutes of Health (NIH) consensus conference recommended that women at risk of preterm birth before 34 weeks of gestation, who delivered within 7 days, should routinely be given a course of ACS [3]. Since then, the incidence and mortality rates of RDS, intraventricular hemorrhage, and necrotizing enterocolitis in preterm infants have been significantly reduced.

However, corticosteroid therapy has some adverse maternal effects, including adrenal suppression and altered glucose tolerance [4]. Mathiesen [5] reported that diabetic mothers receiving glucocorticoid treatment for fetal lung maturation may have poor glycemic control and need to adjust insulin usage. To what extend short-term use of corticosteroids for fetal lung maturation affects fasting blood glucose and insulin levels in normal singleton pregnancies, normal twin pregnancies, and IGT pregnancies who do not require insulin treatment is still uncertain. In this study, we investigated whether glucose and insulin levels differ after the administration of dexamethasone among singleton and twin pregnancies and in pregnancies with impaired glucose tolerance (IGT) [6].

## 2. Materials and Methods

Fifty-six women aged between 25 and 35 years old who received dexamethasone therapy for fetal lung maturation between 28 and 33 weeks of gestation at the Women's Hospital School of the Medicine Zhejiang University from Jan 2009 to Jan 2011 were recruited into this study. All of them had received oral glucose tolerance test (OGTT) [6] between 24 and 28 weeks to screen for gestational diabetes. Exclusion criteria included gestational hypertension, diabetes, renal disorders, and infectious diseases. Patients were divided into three groups: group I (singleton pregnancies) 22 people, group II (twin pregnancies) 18 people, and group III (singleton pregnancies with IGT) 16 people. All of the women gave their informed consent prior to inclusion into the study, which was approved by the Ethics Committee of Zhejiang University.

Dexamethasone usage was according to the Guideline by Obstetrics group of Chinese Medical Association [7]; the women with singleton pregnancies received four doses of 5 mg dexamethasone intramuscularly every 12 h; women with twin pregnancies received six doses of 5 mg dexamethasone intramuscularly every 8 h. Fasting blood samples were collected before the dexamethasone therapy was commenced in the morning and 24 h, 48 h, and 72 h after treatment was completed. Plasma glucose and insulin levels were then detected.

SPSS 11.5 statistical software was used for statistical analysis. All data were expressed as mean ± standard deviation. The F-test was used to determine statistically significant differences between the groups. A *P* value of <0.05 was considered to be statistically significant.

## 3. Results

There was no significant difference in terms of age, gestational weeks, and prepregnancy body mass index (BMI; Table 1). The blood glucose level in group I was significantly lower than that in groups II and III 24 h and 48 h after dexamethasone therapy was started (*P* < 0.01). However, there was no significant difference before treatment was started and 72 h after treatment (*P* > 0.05; Table 2). The insulin level in group III was significantly higher than that in groups I and II before treatment was started and 72 h after treatment (*P* < 0.05); 24 h and 48 h after dexamethasone therapy, the insulin levels in group I were significantly lower than those in groups II and III (*P* < 0.01). There was no difference in the insulin level between groups II and III (*P* > 0.05; Table 3).

## 4. Discussion

In this study, we found that the fasting blood glucose levels 24 h and 48 h after the first injection of dexamethasone in groups II and III were significantly higher than those in group I (*P* < 0.01), but the difference of blood glucose levels among all three groups either before treatment or 72 h afterwards was not significant. Since the biologic half-life of dexamethasone is approximately 36 h to 54 h [8], this indicates that blood glucose levels elevation in groups II and III were consistent with elevated concentration of dexamethasone after injection.

In twin pregnancies, perhaps due to the enlarged placenta, more insulin antagonistic hormones are secreted, such as glucocorticoids, placental lactogen, progesterone, estrogen, and others. In IGT women, exceed insulins were secreted to maintain normal glucose levels although the baseline blood glucose level in groups II and III in our study was within the normal reference range before dexamethasone administration. In these groups, insulin secretion by the pancreatic *β*-cells was in a “full load” state due to the increased levels of insulin antagonistic hormones. After dexamethasone was given to promote fetal lung maturation, insulin resistance increased, which meant that *β*-cell secretion was not sufficient to offset the insulin antagonistic hormones. This was the most likely mechanism that led to the resultant hyperglycemia.

Determination of insulin levels before and after dexamethasone injection showed that the insulin levels before and 72 h after the injections in group III were significantly greater than those of groups I and II, which indicated an increased absolute insulin value and an insulin resistance state in women with IGT. The blood glucose and the insulin levels 24 h and 48 h after dexamethasone administration in groups II and III were both synchronized with the corresponding values in group I. Foglia et al. [9] have suggested that dexamethasone has different effect on maternal glucose levels in twin and singleton pregnancies. Within 24 h of the first corticosteroid injection, twin pregnancies had a higher average maternal glucose levels than singleton pregnancies. This may be due to a higher rate of abnormal glucose tolerance in mothers with twin pregnancies than singleton pregnancies. In addition, Yang et al. [10] found that the use of dexamethasone in the final trimester of pregnancy could increase insulin resistance. Pregnant women with normal glucose tolerance have normal pancreatic islet *β*-cell function. Insulin secretion is increased through enhancement of pancreatic islet *β*-cell function. Consequently, the antagonistic effect of dexamethasone is ameliorated, so that blood glucose levels remain within a normal narrow range. However, the insulin secretion of pregnant women with IGT is significantly increased under normal circumstances. After the administration of dexamethasone, these women are unable to compensate for the action of corticosteroids by enhancing islet *β*-cell function, which results in the increased blood glucose levels.

In conclusion, promoting fetal lung maturation with dexamethasone affects maternal glucose metabolism. The degree of modification of the maternal fasting plasma glucose and insulin levels is correlated with the basic maternal glucose metabolic situation and may correlated with twin pregnancy status. Consequently, blood glucose levels of twin pregnancies and those with impaired glucose metabolism should be closely monitored during the use of dexamethasone for fetal lung maturation.

## Figures and Tables

**Table 1:** Demographic characteristics (*x* ± *s*).

Group	Number of people	Age (years)	Gestational age (weeks)	BMI
I	22	27.1 ± 4.2	30.4 ± 3.1	22.4 ± 3.2
II	18	29.3 ± 2.8	31.2 ± 2.4	21.7 ± 2.9
III	16	27.2 ± 3.4	32.8 ± 2.7	23.4 ± 2.8

**Table 2:** Blood glucose levels before and after dexamethasone therapy (*x* ± *s* mmol/L).

Group	Before treatment	24 h	48 h	72 h
I	4.1 ± 1.9	5.3 ± 2.2*	5.4 ± 1.6*	4.9 ± 2.1
II	3.8 ± 2.2	6.9 ± 2.5	7.3 ± 1.9	4.7 ± 1.9
III	4.2 ± .4	7.3 ± 2.4	8.1 ± 3.1	5.2 ± 2.1

*Group I compared with group II and group III, *P < 0.01.

**Table 3:** Blood insulin levels before and after dexamethasone therapy (*x* ± *s* mmol/L).

Group	Before treatment	24 h	48 h	72 h
I	8.4 ± 2.1	45.3 ± 17.2**	56.2 ± 21.4**	6.1 ± 2.8
II	9.0 ± 3.2	68.8 ± 21.5	98.3 ± 31.9	8.2 ± 3.0
III	19.2 ± 3.4*	76.9 ± 22.4	106.1 ± 33.1	20.2 ± 4.4*

*Group III compared with group I and group II, *P < 0.05; **group I compared with group II and group III, **P < 0.01.
